# Induced Inflammatory and Oxidative Markers in Cerebral Microvasculature by Mentally Depressive Stress

**DOI:** 10.1155/2023/4206316

**Published:** 2023-02-18

**Authors:** Yuequan Zhu, Yazeed Haddad, Ho Jun Yun, Xiaokun Geng, Yuchuan Ding

**Affiliations:** ^1^China-America Institute of Neuroscience, Beijing Luhe Hospital, Capital Medical University, China; ^2^Department of Neurosurgery, Wayne State University School of Medicine, USA; ^3^Department of Neurology, Beijing Luhe Hospital, Capital Medical University, China

## Abstract

**Background:**

Cerebrovascular disease (CVD) is recognized as the leading cause of permanent disability worldwide. Depressive disorders are associated with increased incidence of CVD. The goal of this study was to establish a chronic restraint stress (CRS) model for mice and examine the effect of stress on cerebrovascular inflammation and oxidative stress responses.

**Methods:**

A total of forty 6-week-old male C57BL/6J mice were randomly divided into the CRS and control groups. In the CRS group (*n* = 20), mice were placed in a well-ventilated Plexiglas tube for 6 hours per day for 28 consecutive days. On day 29, open field tests (OFT) and sucrose preference tests (SPT) were performed to assess depressive-like behaviors for the two groups (*n* = 10/group). Macrophage infiltration into the brain tissue upon stress was analyzed by measuring expression of macrophage marker (CD68) with immunofluorescence in both the CRS and control groups (*n* = 10/group). Cerebral microvasculature was isolated from the CRS and controls (*n* = 10/group). mRNA and protein expressions of tumor necrosis factor-*α* (TNF-*α*), interleukin-1*β* (IL-1*β*), interleukin-6 (IL-6), vascular cell adhesion molecule-1 (VCAM-1), and macrophage chemoattractant protein-1 (MCP-1) in the brain vessels were measured by real-time PCR and Western blot (*n* = 10/group). Reactive oxygen species (ROS), hydrogen peroxide (H_2_O_2_), and nicotinamide adenine dinucleotide phosphate (NADPH) oxidase (NOX) activities were quantified by ELISA to study the oxidative profile of the brain vessels (*n* = 10/group). Additionally, mRNA and protein expressions of NOX subunits (gp91^phox^, p47^phox^, p67^phox^, and p22^phox^) in the cerebrovascular endothelium were analyzed by real-time PCR and Western blot (*n* = 10/group).

**Results:**

CRS decreased the total distances (*p* < 0.05) and the time spent in the center zone in OFT (*p* < 0.001) and sucrose preference test ratio in SPT (*p* < 0.01). Positive ratio of CD68^+^ was increased with CRS in the entire region of the brain (*p* < 0.001), reflecting increased macrophage infiltration. CRS increased the expression of inflammatory factors and oxidative stress in the cerebral microvasculature, including TNF-*α* (*p* < 0.001), IL-1*β* (*p* < 0.05), IL-6 (*p* < 0.05), VCAM-1 (*p* < 0.01), MCP-1 (*p* < 0.01), ROS (*p* < 0.001), and H_2_O_2_ (*p* < 0.001). NADPH oxidase (NOX) was activated by CRS (*p* < 0.01), and mRNA and protein expressions of NOX subunits (gp91^phox^, p47^phox^, p67^phox^, and p22^phox^) in brain microvasculature were found to be increased.

**Conclusions:**

To our knowledge, this is the first study to demonstrate that CRS induces depressive stress and causes inflammatory and oxidative stress responses in the brain microvasculature.

## 1. Introduction

Cerebrovascular diseases (CVD) are conditions that narrow blood flow and the blood vessels in the brain, with stroke being the most important and devastating clinical manifestation of CVD [1]. CVD is the second most common cause of death and one of the top five causes of morbidity worldwide, causing a huge burden on patients and their families [2, 3]. Common risk factors for CVD include family history, hyperlipidemia, hypertension, and hyperglycemia [4].

Additionally, psychological stress has been recognized as a risk factor, which includes work-related stress, psychological distress, depression, anxiety, and negative personality traits, namely, anger or hostility [5–7]. It has been found that approximately 40% of CVD cases are associated with psychological distress [8]. Psychological factors are related to increased incidence of death due to CVD [9].

A correlation has been found between mental stress and the incidence of myocardial infarction, stroke, and coronary heart disease (CHD) [10]. Numerous clinical guidelines explain that psychosocial support plays a vital role in the rehabilitation for patients suffering from cardiovascular disease [11–13]. Most studies on psychological stress-induced vascular diseases primarily focus on cardiovascular diseases; consequently, the effects and pathophysiology of psychological stress on CVD remains largely unknown [10].

Inflammation and oxidative stress play a vital role in the progression of CVD [14–17]. Psychological stress is found to increase inflammation [18] and oxidative stress responses [19], additionally it appears that cerebrovascular inflammation and oxidative stress may be the foundation of mental stress-induced CVD. Nicotinamide adenine dinucleotide phosphate (NADPH) oxidase (NOX) is a multisubunit enzyme complex which utilizes NADPH to produce superoxide anions and reactive oxygen species (ROS) in the brain [20, 21]. NOX complex contained membrane subunits (gp91^phox^, p22^phox^) and cytosolic subunits (p47^phox^, p67^phox^), and major NOX subunits have been found in the brain. Upregulation of these subunits is associated with increased NOX activity [20, 22]. ROS from NOX is the basis of early and late inflammatory responses in atherosclerosis of the aorta, and inhibition of NOX activity is found to reduce the progression of atherosclerotic lesions [23, 24].

The primary objective of this study was to study the implications of mental stress on CVD mediated by depressive stress-modulated vascular inflammation and oxidative injuries [25]. This study analyzed the effects of depressive stress on vascular inflammation and oxidative injury by applying a well-established depressive-like model in wild-type C57BL/6J mice. The results of this study could improve the understanding of the risk profile in cerebrovascular disease with an emphasis on psychological stressors.

## 2. Materials and Methods

### 2.1. Subjects

**Figure 1:**
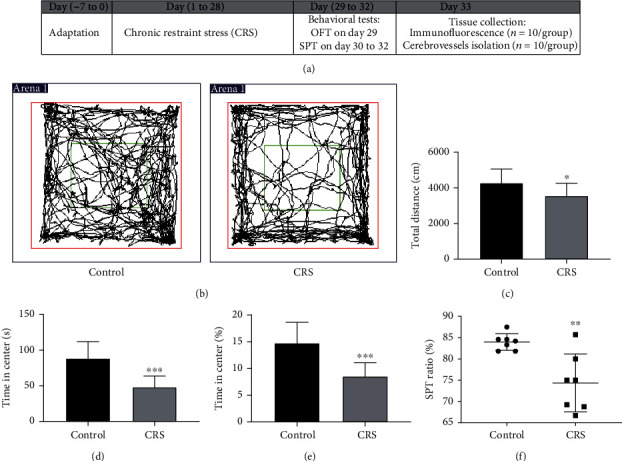
Chronic restraint stress (CRS) induces depressive-like behaviors. (a) Timeline for CRS, validation by behavioral tests, and tissue collection. (b) Representative images of running paths in the OFT. (c) The total running distances were decreased upon CRS. (d, e) The time in the center zone and the percentage of time in the center zone were decreased significantly. (f) CRS could induce a decrease in the SPT ratio. Values are mean ± SE. ^∗^*p* < 0.05, ^∗∗^*p* < 0.01, and ^∗∗∗^*p* < 0.001, as compared with the control group.

The animal research protocol in this study was consistent with the NIH Guide for the Care and Use of Laboratory Animals and approved by the Animal Care and Use Committee of Capital Medical University in Beijing, China. A total of forty 6-week-old adult male C57BL/6J mice (18-22 g) from the Vital River Laboratory Animal Technology Co. Ltd. (Beijing, China) were randomly grouped into the chronic restraint stress (CRS) (*n* = 10/group × 2) and control groups (*n* = 10/group × 2). All mice were housed in an environment with a 12-hour dark/light cycle, controlled temperature (22 ± 2°C), and humidity. Mice were provided with unlimited access to water and food.  Figure 1(a) illustrates the timeline along which CRS, validation using the open field tests (OFT), sucrose preference test (SPT), and tissue collection were performed.

### 2.2. Chronic Restraint Stress (CRS)

CRS was performed as described in the preceding study [25]. Twenty mice in the CRS groups were placed in well-ventilated Plexiglas tubes (inner diameter, 6 cm) without food and water for 6 hours per day (from 09:00 to 15:00 every day) for 28 consecutive days. The control animals were handled for 5 minutes in the same manner as the CRS group and kept in their cages without food or water. OFT and SPT were then performed starting from day 29.

### 2.3. Open Field Tests (OFT)

Ten mice were randomly selected from each group and examined for OFT. OFT has been reported as the most sensitive test for environmental factors [26]. They were conducted to measure the depressive changes 24 hours after the last stress session (day 29). Mice were placed in an open-field apparatus (50 × 50 × 50 cm) to measure anxiety-like behaviors. Their behaviors were monitored for 10 minutes, using a digital camera. The images were captured by an IBM computer with SMART 3.0 animal behavior recording and analysis system (Panlab, Spain). The running paths, total distances traveled, and the time spent in the center zone (12.5 × 12.5 cm) were calculated.

### 2.4. Sucrose Preference Test (SPT)

Ten mice were randomly selected from both groups and tested for SPT. SPT was performed as described in the previous study [25]. Mice were acclimated to 1% sucrose solution (*w*/*v*) for 24 hours with two bottles of 1% sucrose solution in each cage on day 30. On the second day, a bottle of 1% sucrose solution and a bottle of water were provided for another 24 hours. The sucrose and water bottles were then placed in randomly assigned sides of the cage. Following a 12-hour period of food and water deprivation, mice were given the sucrose solution and water for 24 hours. Consumption of water and sucrose during the last 24-hour period was measured. Sucrose preference ratio was calculated according to the following formula:

(1) $$\begin{array}{c} \text{Sucrose preference ratio }\left(\%\right)=\text{sucrose intake }\left(\text{g}\right)/\left[\text{sucrose intake}\left(\text{g}\right)+\text{water }\left(\text{g}\right)\right]\times 100\left(\%\right). \end{array}$$

### 2.5. Immunofluorescence of CD68

After the behavioral tests, ten mice from each group were selected randomly and a total of twenty mice were used for immunofluorescence assay to study the cellular expression of CD68 [27]. The brain tissues were dehydrated, embedded in paraffin, and prepared in 5 *μ*m sections. The sections were deparaffinized with xylene, rehydrated, and washed with phosphate-buffered saline (PBS) for 5 minutes three times. Antigen retrieval was achieved, using a 10 mM citrate buffer (pH = 6.0) for 15 minutes at 96°C. All sections were blocked in 2% milk for 1 hour and probed with the anti-CD68 antibodies (1 : 1000, ab237968, Abcam) overnight at 4°C. After washing, the sections were incubated for 2 hours at room temperature and exposed to anti-rat/anti-rabbit IgG secondary antibody (ZB-2301, 1 : 500, Beijing ZhongShan Inc.). Cellular CD68 expression was analyzed using a computer-assisted microscope (Neurolucid™, MicrobrightWled, USA) and an image analysis system. Quantitative analysis was conducted by randomly counting CD68-immunolabeled cells under a light microscope at 400x magnification throughout 24 nonoverlapping regions of the brain tissue (0.025 mm^2^ each) [26]. The positive cell ratio was calculated for all representative images.

### 2.6. Isolation of Microvasculature of the Entire Brain

After the behavioral tests, ten mice from each group were sacrificed and microvasculature of the whole brain was isolated as performed in the previous studies [28, 29]. The brain was removed after cardiac perfusion, homogenized in a 3 mL ice-cold sucrose buffer (0.32 mol/L sucrose, 3 mmol/L HEPES, and pH 7.4), and centrifuged at 4°C for 10 minutes at 1000 g. The supernatant was discarded, and the pellet was resuspended once again in 3 mL of cold sucrose buffer on ice, homogenized, and centrifuged at 4°C for 10 minutes at 1000 g. The sediment was resuspended in a sucrose buffer and centrifuged twice for 30 seconds at 100 g. The pellets were, then, pooled and washed twice with a sucrose buffer and once with phosphate-buffered saline+0.1% bovine plasma albumin at 200 g. The final pellet was suspended in 1.0 mL of phosphate-buffered saline+0.1% bovine plasma albumin and centrifuged at 14,000 g, and the precipitate was stored at −70°C.

### 2.7. mRNA Expression in Microvasculature

The expression of mRNA of the isolated cerebral vessels was detected, using PCR as previously described [29]. Isolated vessels were homogenized (*n* = 10/group), and RNA was isolated with the TRIzol reagent (Invitrogen). Total RNA was converted into cDNA with the High-Capacity cDNA Reverse Transcription Kit (Applied Biosystems). Quantification of gene expression was performed by the Prism 7500 real-time PCR system (Applied Biosystems). All reactions were performed under the following conditions: 95°C for 15 minutes, 40 cycles of 95°C for 10 seconds, and 60°C for 32 seconds. *β*-Actin was used as the control gene, and all data were recorded as fold differences from *β*-actin level. The primers for mouse, including TNF-*α*, IL-6, IL-1*β*, VCAM-1, MCP-1, gp91^phox^, p47^phox^, p67^phox^, p22^phox^, and *β*-actin, were recorded as shown in Table 1.

### 2.8. Protein Expression in Microvasculature

Western blotting was used to detect protein expression in the cerebrovascular endothelium as performed in the past study [29]. Isolated brain vessels were homogenized and processed, and protein concentrations were measured with a bicinchoninic acid protein assay kit (Pierce Biotechnology, Inc.). Primary antibodies to tumor necrosis factor-*α* (TNF-*α*) (1 : 1000, 11948, Cell Signaling Technology), interleukin-1*β* (IL-1*β*) (1 : 5000, ab9722, Abcam), interleukin-6 (IL-6) (1 : 200, ab7737, Abcam), vascular cell adhesion molecule-1 (VCAM-1) (1 : 2000, ab134047, Abcam), macrophage chemoattractant protein-1 (MCP-1) (1 : 1000, ab25124, Abcam), gp91^phox^ (1 : 1000, ab129068, Abcam), p47^phox^ (1 : 1000, sc-17845, Santa Cruz, Inc.), p67^phox^ (1 : 1000, sc-374510, Santa Cruz, Inc.), p22^phox^ (1 : 1000, sc-271968, Santa Cruz, Inc.), and *β*-actin (1 : 5000, A5060, Sigma-Aldrich) were incubated on the membrane at 4°C overnight. The membranes were washed three times with PBS for 10 minutes each and reincubated with secondary antibody (1 : 10,000, Invitrogen) for 1 hour at room temperature. Protein expressions were analyzed with an enhanced chemiluminescence kit (Millipore). Quantification of relative target protein expression was performed by ImageJ 1.42 (National Institutes of Health).

### 2.9. Expression of ROS and H_2_O_2_ in Microvasculature by ELISA

Mouse enzyme-linked immunosorbent assay (ELISA) kit was used in accordance with the manufacturer's instructions to quantify the expression of reactive oxygen species (ROS) (ml009876-1, MLBIO) and hydrogen peroxide (H_2_O_2_) (ml076343, MLBIO) in the isolated brain microvasculature [29]. Isolated microvasculature was homogenized and washed with saline, and the supernatant was collected.

### 2.10. NADPH Oxidase (NOX) Activity

NOX activity was assessed as described previously [30]. The brain microvasculature samples containing phenylmethylsulfonyl fluoride and protease inhibitor cocktail (Thermo Fisher Scientific; 20 *μ*L) were added to a 96-well luminescence plate containing 6.25 *μ*mol/L of lucigenin. The reaction was initiated by an addition of nicotinamide adenine dinucleotide phosphate (100 *μ*mol/L). NOX activity was measured by the change in luminescence recorded by the DTX-880 Multimode Detector.

### 2.11. Statistical Analysis

Data were expressed as mean values ± SE. The differences between the mean values of two groups were calculated by Student's *t*-test, followed by post hoc comparisons, using the unpaired comparison test. All analyses were performed by GraphPad Prism v5.0 (GraphPad Software, San Diego, CA). In all cases, *p* < 0.05 was considered statistically significant.

## 3. Results

### 3.1. CRS Induced Depressive-Like Behaviors

Images of running paths of open field test (OFT) after CRS were shown in Figure 1(b). Ten mice were randomly selected from each group, and a total of twenty mice were examined for OFT. CRS decreased the total running distances to 3561 ± 704 cm as compared to the control (4280 ± 785 cm, *p* < 0.05; Figure 1(c)). The time spent in the center zone was decreased to 48 ± 15 seconds (8.6 ± 2.5%) versus 88 ± 23 seconds from the control group in 10-minute tests (14.8 ± 3.9%, *p* < 0.001; Figures 1(d) and 1(e)). The sucrose preference test ratio was decreased from 84 ± 1.9% to 74 ± 6.8% (*p* < 0.01, Figure 1(f)). These results indicated that CRS induces depressive-like behaviors.

### 3.2. CRS Increased Infiltration of CD68^+^-Positive Cells

**Figure 2:**
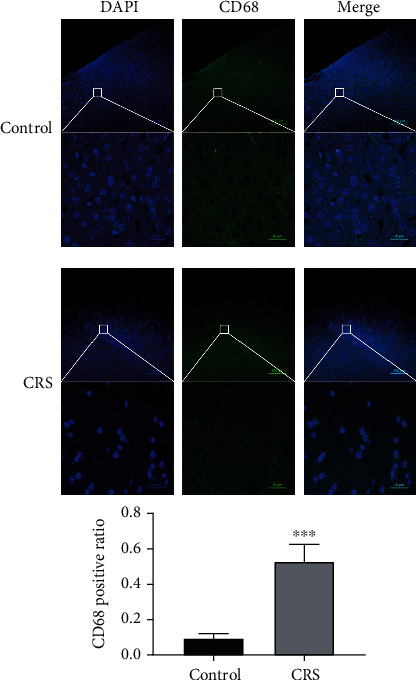
CRS increased infiltration of CD68^+^-positive cells. Immunofluorescence staining of CD68^+^ showed that CRS could induce a larger number of macrophage infiltration. The figures are shown at 200x and 1000x magnification. Values are mean ± SE. ^∗∗∗^*p* < 0.001, as compared with the control group.

Ten mice of each group were randomly chosen, and a total of twenty mice were examined for immunofluorescence of CD68. To delineate the influence of CRS on neuroinflammation, the expression of CD68, or a macrophage marker, was examined with immunofluorescence. As compared to the control group, larger numbers of macrophages were observed in the brain, indicating increased macrophage infiltration into the brain tissue after CRS. The CD68^+^-positive ratio was increased to 53 ± 9.6% (versus 9.3 ± 2.6% from the control group) (*p* < 0.001, Figure 2).

### 3.3. CRS and Microvascular Inflammation

**Figure 3:**
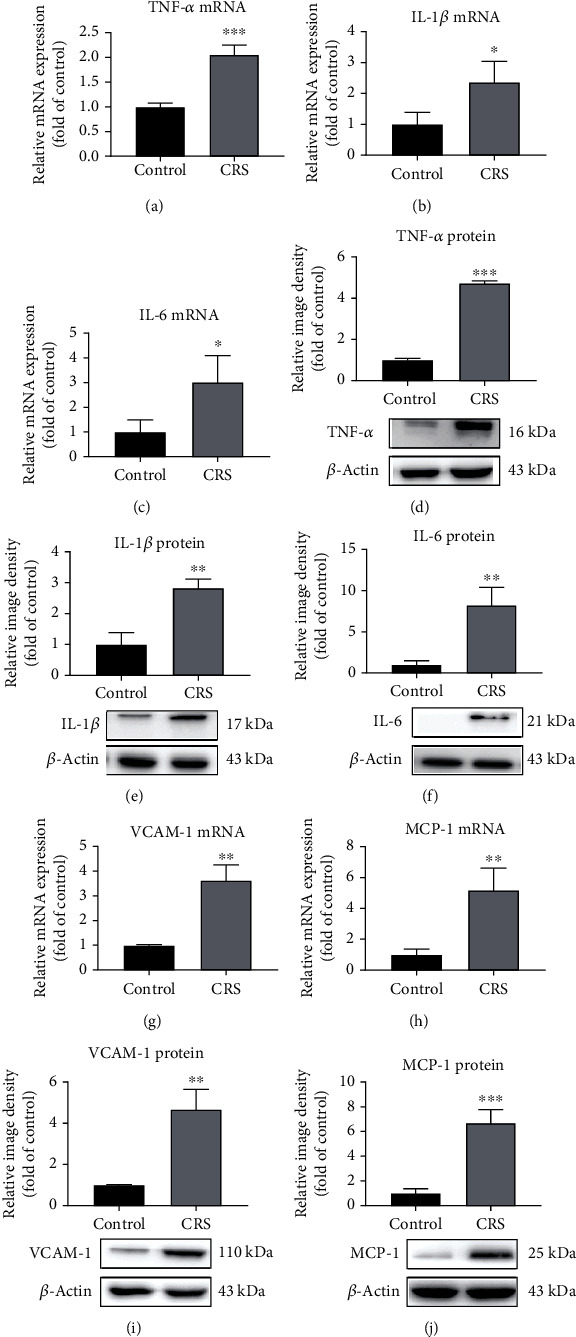
Psychological stress increased cerebrovascular inflammation of mice. (a) mRNA and (d) protein expressions of TNF-*α* were increased after CRS. (b) mRNA and (e) protein expressions of IL-1*β* were increased upon CRS. (c) mRNA and (f) protein expressions of IL-6 were upregulated upon CRS. (g) CRS could cause an increase in the expression of VCAM-1 in mRNA and (i) protein levels. (h) CRS could induce an upregulation of the mRNA and (j) protein expressions of MCP-1. Representative immunoblots are displayed at the top of the graphs. Values are mean ± SE. ^∗^*p* < 0.05, ^∗∗^*p* < 0.01, and ^∗∗∗^*p* < 0.001, as compared with the control group.

Ten mice of each group were examined for microvasculature isolation. The effects of CRS on inflammatory factors in the brain microvasculature were studied with mRNA and protein levels 24 hours after the last session of CRS. mRNA and protein expressions of TNF-*α* were increased to 2.1 ± 0.2-fold (*p* < 0.001, Figure 3(a)) and 4.7 ± 0.1-fold (*p* < 0.001, Figure 3(d)), respectively, in the CRS group. Similarly, mRNA expression of IL-1*β* and IL-6 was upregulated to 2.4 ± 0.7-fold (*p* < 0.05, Figure 3(b)) and 3 ± 1-fold (*p* < 0.05, Figure 3(c)) after CRS and the protein expression was increased to 2.8 ± 0.3-fold (*p* < 0.01, Figure 3(e)) and 8.2 ± 2.2-fold (*p* < 0.01, Figure 3(f)), respectively. As shown in Figures 3(g) and 3(i), there was a clear increase in the expression level of VCAM-1 upon CRS. VCAM-1 mRNA expression was increased 3.6 ± 0.6 times (*p* < 0.01, Figure 3(g)) and a similar trend was seen in the protein expression (*p* < 0.01, Figure 3(i)). MCP-1, an important proinflammatory cytokine in atherosclerosis, showed increased expression of mRNA and protein (5.2 ± 1.4 times, *p* < 0.01 and 6.7 ± 1 times, *p* < 0.001), respectively, by CRS (Figures 3(h) and 3(j)).

### 3.4. Increased Oxidative Stress and NOX Activity in the Brain Microvasculature by CRS

**Figure 4:**
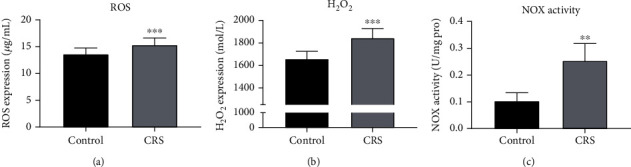
CRS increased oxidative stress and NOX activity in the brain microvasculature of mice. (a) ROS production was increased upon mental stress in the vessels of brains through ELISA. (b) Total H_2_O_2_ in the cerebrovascular endothelial was increased upon CRS. (c) CRS could induce an upregulation in NOX activity. Values are mean ± SE. ^∗∗^*p* < 0.01, ^∗∗∗^*p* < 0.001, as compared with the control group.

When the same brain tissues were further studied, production of ROS (*p* < 0.001, Figure 4(a)) and H_2_O_2_ (*p* < 0.001, Figure 4(b)) were noted to be significantly increased by stress. The activity of NADPH oxidase (NOX), which facilitates the production of ROS and H_2_O_2_, was detected. Increased activity of NOX by CRS is a potential explanation of ROS and H_2_O_2_ production (*p* < 0.01, Figure 4(c)).

### 3.5. Effects of CRS on Expression of NOX Subunits in Microvasculature

**Figure 5:**
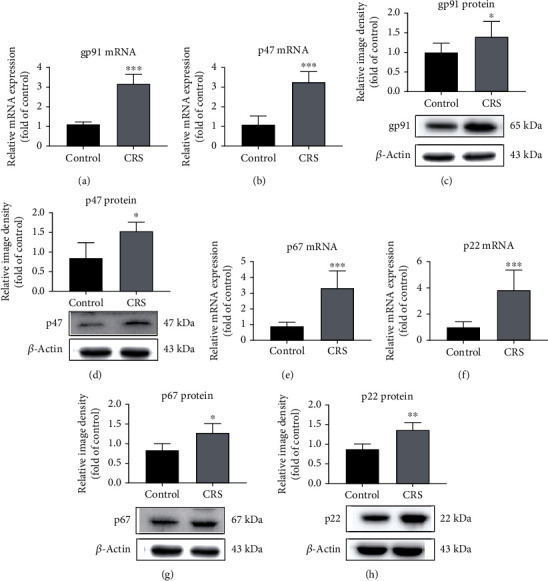
CRS increased the expression of NOX subunits in microvasculature. (a, c) CRS could increase the expression of mRNA and protein of gp91^phox^. (b, d) CRS could promote the expression level of mRNA and protein of p47^phox^. (e, g) mRNA and protein expressions of p67^phox^ were upregulated upon CRS. (f, h) mRNA and protein expressions of p22^phox^ were increased after CRS. Representative immunoblots are displayed under the graphs. Values are mean ± SE. ^∗^*p* < 0.05, ^∗∗^*p* < 0.01, and ^∗∗∗^*p* < 0.001, as compared with the control group.

As above, the effect of CRS on NOX subunits (gp91^phox^, p47^phox^, p67^phox^, and p22^phox^) in the brain microvasculature was analyzed. The mRNA expression of gp91^phox^, p47^phox^, p67^phox^, and p22^phox^ increased 2.9 ± 0.4-fold (*p* < 0.001, Figure 5(a)), 3 ± 0.5-fold (*p* < 0.001, Figure 5(b)), 3.7 ± 1.2-fold (*p* < 0.001, Figure 5(e)), and 3.8 ± 1.5-fold (*p* < 0.001, Figure 5(f)), respectively. Western blot analyses demonstrated the same trend for these subunits. Comparing to the control group, protein expression of gp91^phox^, p47^phox^, p67^phox^, and p22^phox^ increased 1.4 ± 0.4 (*p* < 0.05, Figure 5(c)), 1.8 ± 0.3 (*p* < 0.05, Figure 5(d)), 1.5 ± 0.3 (*p* < 0.05, Figure 5(g)), and 1.6 ± 0.2 times (*p* < 0.01, Figure 5(h)), respectively.

## 4. Discussion

Previous studies regarding the effects of psychological stress on vascular disease primarily focused on the cardiovascular system with use of high-fat diet or apolipoprotein E -/- (ApoE-/-) mice [31, 32]. There are limited studies available addressing the effects of psychological stress on cerebrovascular diseases. The purpose of this study was to investigate the effects of psychological stress on cerebrovascular vessels in wild-type mice with normal diet, using a well-established psychological stress model, namely, CRS.

It has been acknowledged that mental stress induces microglia activation, especially in the hippocampus and prefrontal cortex [33]. Activated microglia and neuroinflammation have been noted from repeated social defeat stress (RSDS) [1]. CD68 is a transmembrane glycoprotein highly expressed in activated and phagocytic microglia [34]. Increased expression of CD68 reflects the activation of microglia [35, 36]. Stress from chronic social defeat [37] or chronic mild stress [38] are found to increase CD68*^+^* microglia and promote their activation. A recent study also reports that CD68 is enriched in the plaque shoulder and necrotic core of atherosclerotic lesions from the human carotid artery [39].

Animal experiments have proved excess inflammatory responses and oxidative injuries in cardiovascular systems from mental stress. Endothelial inflammation is noted to be the initial step in atherosclerosis [40, 41], and cerebrovascular inflammation has been found to play an important role in intracranial atherosclerosis [29, 42, 43] and CVD [44, 45]. Studies have confirmed the involvement of inflammatory mediators in the early proatherogenic processes, such as upregulation of adhesion molecules and highly activated inflammatory responses on endothelial cells [46]. TNF-*α*, IL-6, IL-1*β*, VCAM-1, and MCP-1 play critical roles in regulating vascular inflammation, and increased numbers of these inflammatory factors in brain vessels indicate vascular dysfunction [29, 47].

Mental stress exacerbates vascular inflammation (i.e., TNF-*α*, CRP, MCP-1, and ICAM-1) and decreases endothelial nitric oxide syntheses [48]. Stress-induced vascular inflammation ultimately results in plaque destabilization in atherosclerosis [49]. Stress-induced hyperactivation of hypothalamic-pituitary-adrenal (HPA) and sympathetic-adrenal-medullary (SAM) axes can influence the vascular endothelium function as well as the recruitment of circulating monocytes and their conversion to foam cells [50, 51]. Lehmann et al. [52] describes different gene expressions of the brain endothelial cells (bECs) upon mental stress (i.e., unpredictable chronic mild stress (UCMS)); the results show that stress could cause gene expression changes in inflammation, oxidative stress, growth factor signaling, and wound healing in bECs. Although the findings have not been validated by molecular biology studies, the study utilized the CRS model, one of the most effective and commonly used depressive model [25, 53, 54], and demonstrated increased mRNA and protein expressions of inflammatory factors in the brain vessels, including TNF-*α*, IL-1*β*, IL-6, VCAM-1, and MCP-1.

A few mechanisms of disease have been proposed to explain the impact of mental stress on cerebrovascular inflammation and oxidative injuries. One study suggests that CRS may decrease the size of cerebral vessels and increases blood-brain barrier (BBB) permeability, which eventually promotes leakage of plasma immunoglobulins across the BBB into perivascular and parenchymal spaces [55]. We have reported that repeated social defeat stress could induce microglia activation [1], which subsequently causes cerebrovascular inflammation [56, 57]. In addition, mental stress is found to elevate levels of circulating proinflammatory cytokines and glucocorticoids, which potentially impacts the brain vessels by altering the central inflammatory state via glucocorticoid feedback to the brain [52].

As this study shows, oxidative stress occurs with cerebrovascular inflammation. Oxidative injuries have been shown to play an important role in pathophysiology of CVD as it contributes to endothelial cell dysfunction, monocyte/macrophage recruitment and activation, and ultimately vascular inflammation [58, 59]. Brooks et al. have found increased ROS and reduced NO bioavailability in the MCA of mice with mental stress, and mental stress (UCMS) could impair endothelial-dependent dilation (EDD) and exaggerate vascular constriction [60].

Although there are many potential sources of ROS, NOX has been considered the main contributor. NOX is a multisubunit enzyme complex formed by cytosolic subunits (p47^phox^ and p67^phox^) translocated to membrane subunits (p22^phox^ and gp91^phox^) to function as a catalytic unit. NOX activation has been found in many diseases, such as neurodegenerative diseases, traumatic brain injury (TBI), and stroke. Additionally, inhibition of NOX reduces the development of atherosclerotic plaques in the aorta [23, 61] and improves clinical outcomes from ischemic brain injuries [62], which supports the fact that NOX activation is strongly involved in the pathogenesis of CVD. The findings of this study also demonstrate that NOX activation is involved in the pathogenesis of intracranial atherosclerosis.

Mental stress can induce NOX activation in the endothelium of thoracic aorta [63] and increase the expression of NOX-4 in carotid [64, 65]. This study shows the NOX activation in the brain vessels and the expression of NOX subunits in mRNA and protein levels upon mental stress. This finding suggested that NOX activation may play a vital role in vascular inflammation.

Dyslipidemia induced by a high-fat diet promotes atherosclerotic lesions. In fact, ApoE-/- mice has been widely used for spontaneous atherosclerosis since ApoE-/- mice accumulate high levels of cholesterol in the blood and thus develop atherosclerosis [66]. This study demonstrates mental stress could not only increase the expression of inflammatory factors but also induce oxidative stress responses and NOX activation in cerebrovascular endothelial cells of wild-type C57BL/6J mice with normal diet. These findings suggest that psychological events may play greater roles than lipid disorders or genetic defects in cerebrovascular diseases than thought previously.

## 5. Conclusion

CRS-induced depressive stress instigates the infiltration of macrophage, cerebrovascular inflammation, and oxidative stress responses in the brain. Activation of NOX and the expression of its subunits increase after mental stress. It is very likely that cerebrovascular inflammation and oxidative injuries from psychological stress without high-fat diet serve as an important risk factor for cerebrovascular diseases.

## Figures and Tables

**Table 1:** Primers for real-time polymerase chain reaction (PCR) analysis.

Genes	Forward primer (5′–3′)	Reverse primer (5′–3′)
TNF-*α*	CAGGCGGTGCCTATGTCTC	CGATCACCCCGAAGTTCAGTAG
IL-6	CTGCAAGAGACTTCCATCCAG	AGTGGTATAGACAGGTCTGTTGG
IL-1*β*	GAAATGCCACCTTTTGACAGTG	TGGATGCTCTCATCAGGACAG
VCAM-1	TTGGGAGCCTCAACGGTACT	GCAATCGTTTTGTATTCAGGGGA
MCP-1	CGCCTCCAGCATGAAAGTCT	GGGAATGAAGGTGGCTGCTA
gp91^phox^	AGTGCGTGTTGCTCGACAA	GCGGTGTGCAGTGCTATCAT
p47^phox^	ACACCTTCATTCGCCATATTGC	CCTGCCACTTAACCAGGAACA
p67^phox^	GGAGAAGTACGACCTTGCTATCA	ACAGGCAAACAGCTTGAACTG
p22^phox^	CTACTGCTGGACGTTTCACAC	GGTGGACCCCTTTTTCCTCTT
*β*-Actin	GTGACGTTGACATCCGTAAAGA	GCCGGACTCATCGTACTCC

## Data Availability

The data used to support the findings in this study are available from the corresponding author upon reasonable request.
